# Design and Experimental Evaluation on an Advanced Multisource Energy Harvesting System for Wireless Sensor Nodes

**DOI:** 10.1155/2014/671280

**Published:** 2014-06-16

**Authors:** Hao Li, Gaofei Zhang, Rui Ma, Zheng You

**Affiliations:** Collaborative Innovation Center for Micro/Nano Fabrication, Device and System, State Key Laboratory of Precision Measurement Technology and Instruments, Department of Precision Instrument, Tsinghua University, Beijing 100084, China

## Abstract

An effective multisource energy harvesting system is presented as power supply for wireless sensor nodes (WSNs). The advanced system contains not only an expandable power management module including control of the charging and discharging process of the lithium polymer battery but also an energy harvesting system using the maximum power point tracking (MPPT) circuit with analog driving scheme for the collection of both solar and vibration energy sources. Since the MPPT and the power management module are utilized, the system is able to effectively achieve a low power consumption. Furthermore, a super capacitor is integrated in the system so that current fluctuations of the lithium polymer battery during the charging and discharging processes can be properly reduced. In addition, through a simple analog switch circuit with low power consumption, the proposed system can successfully switch the power supply path according to the ambient energy sources and load power automatically. A practical WSNs platform shows that efficiency of the energy harvesting system can reach about 75–85% through the 24-hour environmental test, which confirms that the proposed system can be used as a long-term continuous power supply for WSNs.

## 1. Introduction

Wireless sensor networks have been widely used for industrial productions, military defense, and disaster monitoring [1]. Since the power supply system for WSNs has a limited capacity and has to be periodically recharged when the batteries run out [2], some key problems of the power supply have not been perfectly resolved. Thus, one of the important demands for a sustainable power supply system of WSNs is to collect energy from ambient environment without compulsory maintenance. Energy harvesting system has been studied extensively by using photovoltaic modules to increase the autonomy of WSNs in recent years due to its large power density [3–5]. In addition, other methods of energy collection have also been investigated, such as pure collection of vibration-based energy source [6–11], wind-based energy source [12], and temperature difference-based energy source [13]. However, the power supply from single energy source is irregular and unpredictable, while multisources energy collection should be considered as an adequate and adjustable system for WSNs.

The first generation of multisource energy harvesting system was presented by Roundy et al. in 2003 [14]. Solar and vibration energy were utilized to power a wireless sensor node, and 3.3 mF capacitor was used to storage the energy. The efficiency of energy collection was not encouraging because power management system or MPPT circuit has not been used. Another multisource energy harvesting system using MPPT circuit was MPWiNodeX [15]. In that system, solar energy, wind energy, and water flow were exploited, but the MPPT circuit was implemented by a MCU, which consumed a large amount of power. The most well-known multisource energy harvesting system in recent years was Ambimax [16]. It used a li-ion battery to perform as backup energy reservoir and included a MPPT circuit as well; however, the power management in that system was not as optimal as the direct connection of the li-ion battery to the load, and the variation of ambient energy may cause a large load voltage fluctuation. Moreover, the total volume of the system was very large, which is not convenient to power WSNs in some situations.

In this paper, an effective multisource energy harvesting system using solar and vibration energy is presented for WSNs. The implementations of analog driving scheme and ultra-low-power components make the power efficiency of MPPT circuits reach beyond 80% for solar energy harvester and 60% for vibration energy harvester. The super capacitor is used to preserve the lithium polymer battery in constant condition for the reduction of current fluctuation caused by the cyclical peak power of the load. Two prototypes have been developed with overall volume not more than 83 cm^3^ and 29 cm^3^, respectively, which provide an advanced solution for the power supply system of WSNs.

## 2. System Architecture

**Figure 1:**
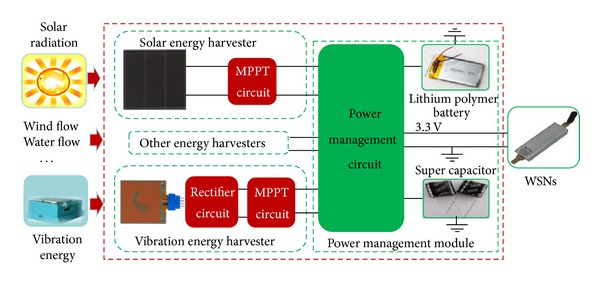
Architecture of the multisource energy harvesting system.

The architecture of the proposed multisource energy harvesting system is highly modular as shown in Figure 1. It contains the solar energy harvester, the vibration energy harvester, and the power management module. A monocrystalline silicon solar panel and a cantilever piezoelectric generator are employed as the energy conversion devices, converting solar and vibration energy to electric power. Each energy harvester includes a MPPT circuit, which dynamically adjusts the operational parameters of the energy conversion devices in response to the variations of energy sources so that the output power can be maximized. The rectifier circuit in the vibration energy harvester is used to convert the output current of piezoelectric generator into direct current. In addition, a lithium polymer battery is integrated in the power management module in order to protect the system from the unpredictable shortage of ambient energy. And a super capacitor with lithium battery in parallel provides instantaneous power output. Moreover, this system can be extended by using other possible kinds of energy harvesters as long as the connection between the power management circuit and the energy harvester is joined.

### 2.1. Solar Energy Harvester

Solar energy is the most widespread energy source in natural environment and provides a large power density, making it a suitable choice to power WSNs [17]. As the output characteristics of a solar panel changes nonlinearly with the varied temperature or irradiance conditions, maximizing the efficiency becomes critically important, and the MPPT circuit should be included in the solar energy harvester to find the operating point (voltage and current) at which the solar panel should operate to deliver the highest possible power [18].

**Figure 2:**
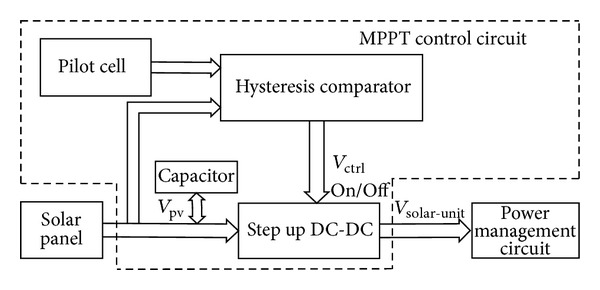
Schematic diagram of solar energy harvester.

Based on our previous work [19], the analog driving MPPT circuit (shown in Figure 2) contains a pilot cell (CPC1824), a capacitor, a hysteresis comparator (LTC1440), and a step up DC-DC converter (LTC3401). When the solar panel is illuminated with sunlight, the capacitor is continuously charged until its voltage is above a particular value, which is the corresponding voltage when the maximized output power is reached. Then the hysteresis comparator generates a control signal to switch on the step up DC-DC converter, transferring the power from the charged capacitor to the power management circuit. As a result, the voltage of the capacitor drops to the lower bound of the hysteresis comparator, causing the step-up DC-DC converter to be switched off, and the capacitor is charged again. The detail principle and implementation of the circuit are present in [19] before this work.

### 2.2. Vibration Energy Harvester

A vibrating piezoelectric element electrically behaves as a capacitive AC source [20, 21]. It should be rectified at a desired DC voltage level for the power supply to an electronic device. Traditional vibration energy harvesting circuit contains the full bridge (FB) diode rectifier with electronic control circuits [22, 23]. It only works effectively in nonadaptive circuits so that it is unable to adjust the operational parameters of the piezoelectric generator for maximizing the output power. In order to overcome the instability of the vibration environment, the voltage double (VD) rectifier and the MPPT circuit are used for the vibration energy harvester in this system.

**Figure 3:**
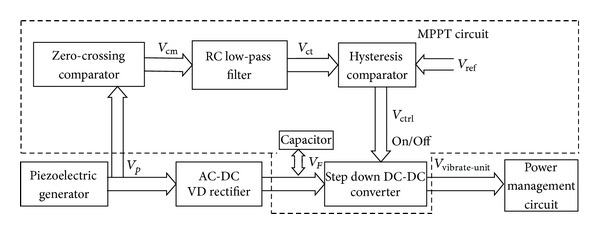
Schematic diagram of the vibration energy harvester.

Figure 3 shows the schematic diagram of the vibration energy harvester. The design of MPPT circuit is based on the theoretical model [24]. In that model, the maximum extracted power of a piezoelectric generator with a VD rectifier can be expressed in

(1) $$\begin{array}{c} {\langle P_{\text{VD}}\rangle }_{\max}=\frac{C_{P}WV_{\text{OC}}^{2}}{2\pi }\;\text{at}\;\;V_{F}=V_{\text{OC}}, \end{array}$$

where *C*_*P*_ denotes the capacitance coefficient of the piezoelectric beam; *V*_OC_ denotes the open circuit voltage of piezoelectric generator; *W* denotes the vibration frequency; and *V*_*F*_ denotes the voltage of piezoelectric generator after rectified.

According to this equation, the maximum extracted power of the piezoelectric generator can be obtained by dynamically adjusting the voltage of capacitor *V*_*F*_ to be equal to that of *V*_OC_ when the vibration conditions change in the ambient environment. To implement the MPPT circuit, several ultra-low-power components are used, including a zero-crossing comparator (LTC1440), an RC low-pass filter, a hysteresis comparator (LTC1440), and a step down DC-DC converter (LTC3632). As shown in Figure 3, the signal generated by the piezoelectric generator is a sine-wave signal (*V*_*P*_). One part of the signal is converted into direct current with the VD rectifier to charge the capacitor; the other part is transformed into the rectangular-wave signal (*V*_cm_) in the zero-crossing comparator. Since only positive part of the sine-wave signal can pass through the zero-crossing comparator, the duty of the rectangular-wave signal is relevant to the voltage of the capacitor (*V*_*F*_). Adjusting the duty of *V*_cm_ can make the value of *V*_*F*_ be equal to that of *V*_OC_ [25]. After passing through a RC low-pass filter, the rectangular-wave signal will be transformed into a rippled DC voltage *V*_ct_ (the average value of *V*_cm_). By comparing the value of *V*_ct_ with *V*_ref_ (the internal reference voltage LTC1440), the hysteresis comparator generates a signal (*V*_ctrl_) to control the step down DC-DC converter to switch on or switch off. If *V*_ct_ rises above *V*_ref_ and reaches the upper bound, the step down DC-DC converter will be switched on and *V*_*F*_ begins to drop. If *V*_ct_ drops below the lower bound of the hysteresis comparator, the step down DC-DC converter will be switched off and *V*_*F*_ begins to increase. With this adjustment, *V*_*F*_ will oscillate around the *V*_OC_, and the vibration energy harvester outputs the possible maximum power.

### 2.3. Power Management Module

The energy reservoirs such as the super capacitor and the rechargeable lithium polymer battery should be required to store the collected power as much as possible since the ambient energy sources are instable. Previous designs [26, 27] attempt to use MCU or DSP to control the charge and discharge process of energy reservoirs. However, most of the collected energy is wasted by the power consumption of the control circuits. In order to provide the effective power management, a p-channel MOSFET (pMOSFET) and three Schottky diodes are used to construct power management circuit.

**Figure 4:**
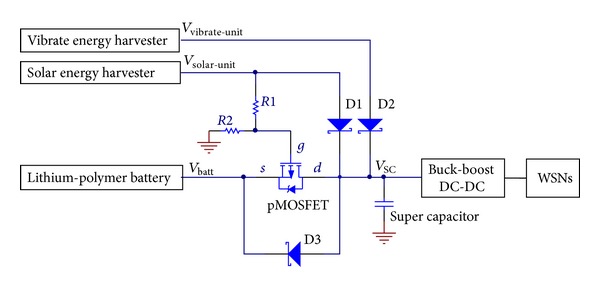
Schematic diagram of power management module.

As shown in Figure 4, the electric potential of the terminals in the pMOSFET can be expressed as (2), where *V*_solar_, *V*_batt_, and *V*_sc_ denote the voltage of solar energy harvester, lithium polymer battery, and super capacitor, respectively. Taking into account the voltage drop caused by the Schottky diodes in Figure 4, in this system, the output voltages of the energy harvesters are set as 4.6 V, which is important to make sure that the energy harvesters can charge the lithium polymer battery when the ambient energy sources are available.

(2) $$\begin{array}{c} V_{g}=V_{\text{solar-unit}}\cdot \frac{R_{2}}{\left(R_{1}+R_{2}\right)},\\ V_{s}=V_{\text{batt}},\\ V_{d}=V_{\text{sc}}. \end{array}$$

The pMOSFET has a threshold voltage (*V*_th_). When the solar energy source is sufficient (*V*_gs_ > *V*_th_), the pMOSFET is switched off, and then the discharge circuit of lithium polymer battery is disconnected. In this case, the energy harvesters charge the super capacitor and power the WSNs. If the voltage difference between *V*_sc_ and *V*_batt_ is larger than forward voltage of the Schottky diode D3, D3 will be conducted so that the lithium polymer battery is charged as well.

Conversely, the pMOSFET is switched on when the solar energy source is insufficient (*V*_gs_ < *V*_th_), and then the discharge circuit of the lithium polymer battery is connected. In this case, if *V*_solar-unit_ < *V*_sc_ and *V*_batt_ > *V*_sc_, the WSNs will be powered by the lithium polymer battery; if *V*_solar-unit_ > *V*_sc_ and *V*_batt_ > *V*_sc_, the lithium polymer battery and solar energy harvester will power the WSNs together. Furthermore, if the vibration energy source is sufficient with *V*_vibrate-unit_ > *V*_sc_, the vibration energy harvester can power the WSNs and charge the lithium polymer battery as well.

## 3. System Evaluation and Prototypes

In order to make sure that the MPPT circuits are valid in tracking the maximum power effectively and examine the role of power management module in the proposed multisource energy harvesting system, in this section, we present the evaluation experiments. Based on the experimental results, prototypes were developed for the proposed system.

### 3.1. Experimental Setup

**Figure 5:**
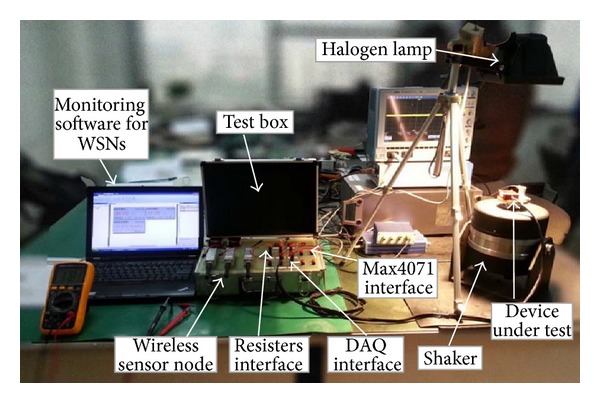
Experimental setup for evaluation tests.

Figure 5 shows the experimental setup for the evaluation of the multisource energy harvesting system. It contains a halogen lamp, a shaker, a test box with a DAQ card (AMUSB-9110), resisters, current sensors (MAX4071), and a wireless temperature sensor node (WTHS-G0). The halogen lamp (PHILIPS QVF135) is used to simulate the sunlight from indoors. The light intensity can be realized by adjusting the distance between the halogen lamp and the solar panel. The light power meter (OPHIR FL250A-LP1-SH-V1) is used to vary the light intensity. In order to simulate the vibration energy harvester, a small shaker (YMC VT-100) is used to vibrate the piezoelectric generator.

### 3.2. Evaluation of the Solar Energy Harvester

Previous work in [19] has demonstrated that the MPPT circuit can properly track the maximum power point. Errors between the real maximum output power of the solar panel and the output power using the MPPT circuit are tested in 0.3%.

**Figure 6:**
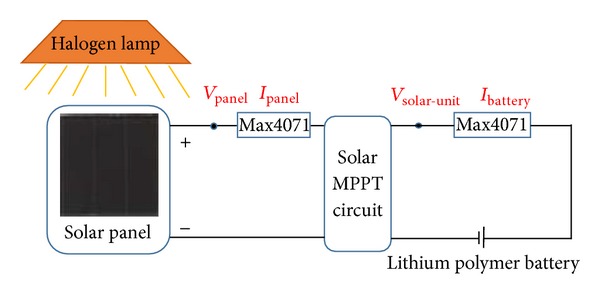
Experimental configuration to evaluate the solar energy harvester.

In this section, the power efficiency of the MPPT circuit (*η*_1_) is evaluated.  Figure 6 shows the experimental configuration. In this experiment, the solar energy harvester is used to charge a lithium polymer battery. Currents are converted into voltages by the current sensors IC (MAX4071). The efficiency *η*_1_ is given in

(3) $$\begin{array}{c} \eta_{1}=\frac{P_{\text{Solar-unit}}}{P_{\text{Panel}}}=\frac{V_{\text{Solar-unit}}\cdot I_{\text{Battery}}}{V_{\text{Panel}}\cdot I_{\text{Panel}}}, \end{array}$$

where *P*_Solar-unit_ denotes the output power of the solar energy harvester; *P*_Panel_ denotes the output power of solar panel; *V*_Panel_ denotes the output voltage of solar panel; *V*_Solar-unit_ denotes the output voltage of solar energy harvester; *I*_Battery_ denotes the charge current of the lithium polymer battery; and *I*_Panel_ denotes the output current of solar panel.

**Figure 7:**
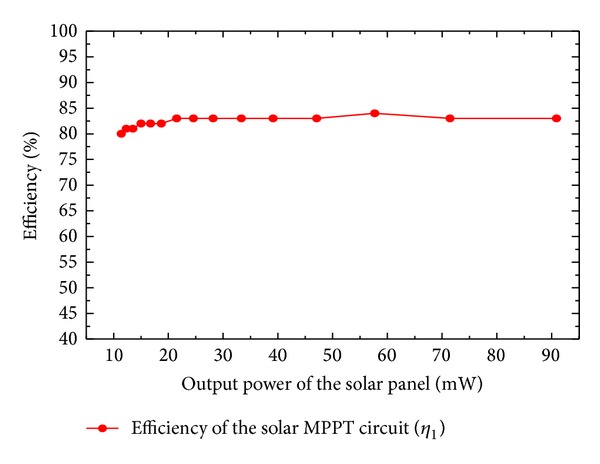
The calculated efficiency of the MPPT circuit for solar energy harvester.

The light intensity can be adjusted between 100 W/m^2^ and 1200 W/m^2^ by altering the distance between the halogen lamp and the solar cell. The results of the calculated efficiency are summarized in Figure 7. This shows that the efficiency of the solar energy harvester can reach more than 80% when the power of solar panel changes from 10 mW to 90 mW. As the hysteresis comparator is powered by the pilot cell CPC1824 in solar energy harvester (see Figure 2), the power losses are mainly caused by the DC-DC converter (LTC3401).

### 3.3. Evaluation of the Vibration Energy Harvester

**Figure 8:**
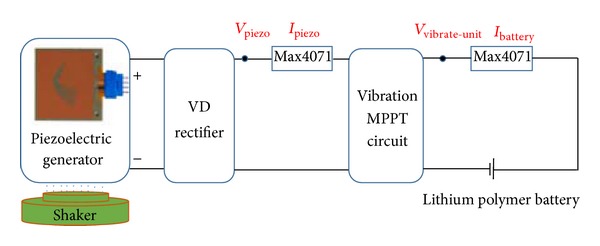
Configuration of the experiment to evaluate the vibration energy harvester.

Figure 8 shows the experimental configuration to evaluate the vibration energy harvester. A piezoelectric generator (Volture V25W) is used as the energy conversion device, which is placed with a 7.8-gram tip mass and made to work in its resonant frequency (44 Hz) with the piezoelectric amplitude varying from 0.5 g to 7 g. For this test, firstly, the MPPT circuit was connected between the VD rectifier and the lithium polymer battery to test the output power and MPPT efficiency (*η*_2_). Then, we removed the MPPT circuit and repeated the test. The efficiency of the *η*_2_ is given in

(4) $$\begin{array}{c} \eta_{2}=\frac{P_{\text{Vibrate-unit}}}{P_{\text{Piezo}}}=\frac{V_{\text{Vibrate-unit}}\cdot I_{\text{Battery}}}{V_{\text{Piezo}}\cdot I_{\text{Piezo}}}, \end{array}$$

where *P*_Vibrate-unit_ denotes the output power of the vibration energy harvester; *P*_Piezo_ denotes the output power of the piezoelectric generator after rectifying; *V*_Piezo_ denotes the output voltage of the piezoelectric generator after rectifying; *V*_Vibrate-unit_ denotes the output voltage of the vibration energy harvester; *I*_Battery_ denotes the charge current of the lithium polymer battery; and *I*_Piezo_ denotes the output current of the piezoelectric generator after rectifying.

**Figure 9:**
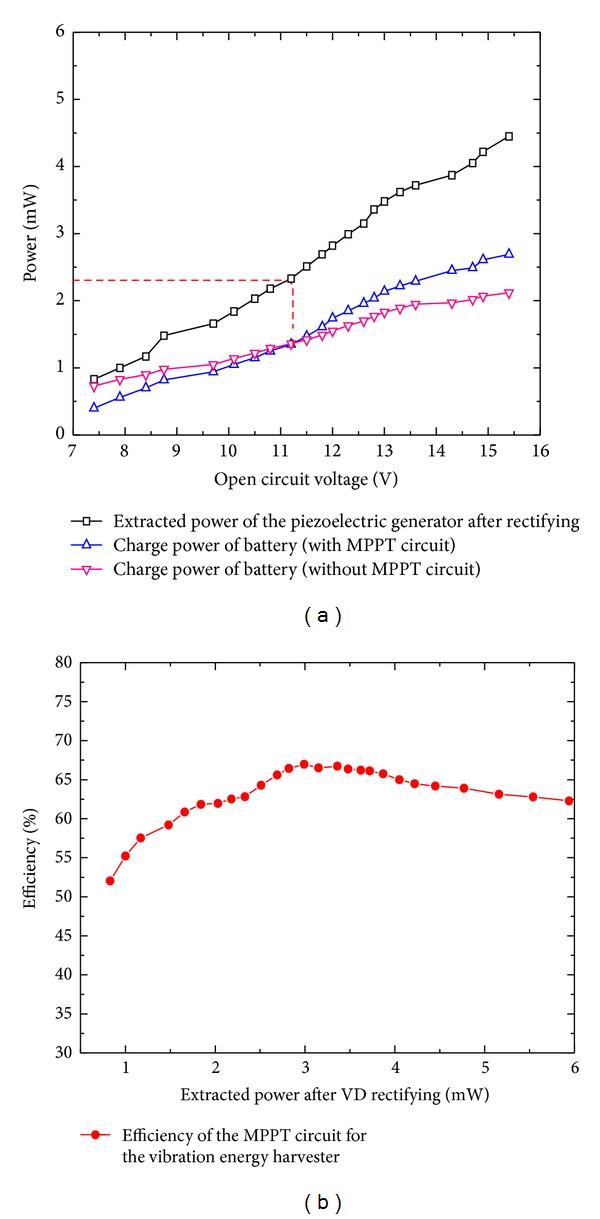
The calculated charge powers of the lithium polymer battery and efficiency of the MPPT circuit. (a) Comparative analysis of the lithium polymer battery charge power. (b) Efficiency of the MPPT circuit.

The results of the calculated output powers and efficiency of the MPPT circuit are summarized in Figure 9, showing that the MPPT circuit plays a vital role in increasing the output power of the vibration energy harvester. The graph in Figure 9(a) compares the charge power of the directly connected lithium polymer battery with the charge power of the lithium polymer battery using the MPPT circuit. From that figure, we can see that, for an extracted power less than 2.3 mW, the charge power using a direct connection is higher than the one with the MPPT circuit due to the power losses caused by the MPPT circuit. However, for an extracted power higher than 2.3 mW, the useful output power of the MPPT circuit rapidly increases and becomes larger than the power of the directly connected test since the MPPT circuit adaptively matches the rectified voltage with the open circuit voltage. The graph in Figure 9(b) shows the efficiency of the MPPT circuit. From this figure, we can conclude that, for an extracted power ranging from 2 mW to 6 mW, the efficiency of MPPT circuit can reach more than 60%. The lost energy in this circuit is mainly caused by the DC-DC converter (LTC3632). With the output power of the piezoelectric generator increasing, the increased voltage difference decreases the efficiency of LTC3632 and results in the slight decrease of the MPPT efficiency.

### 3.4. Evaluation of the Power Management Module

**Figure 10:**
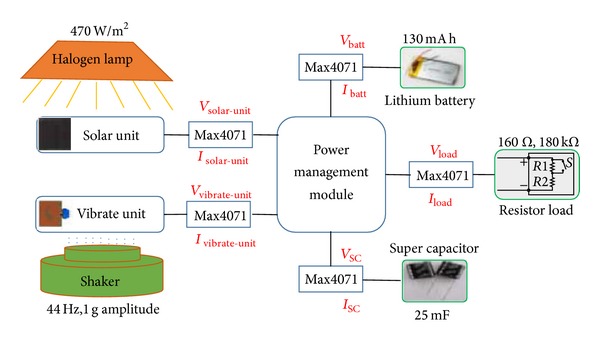
Schematic diagram of the experimental setup.

**Figure 11:**
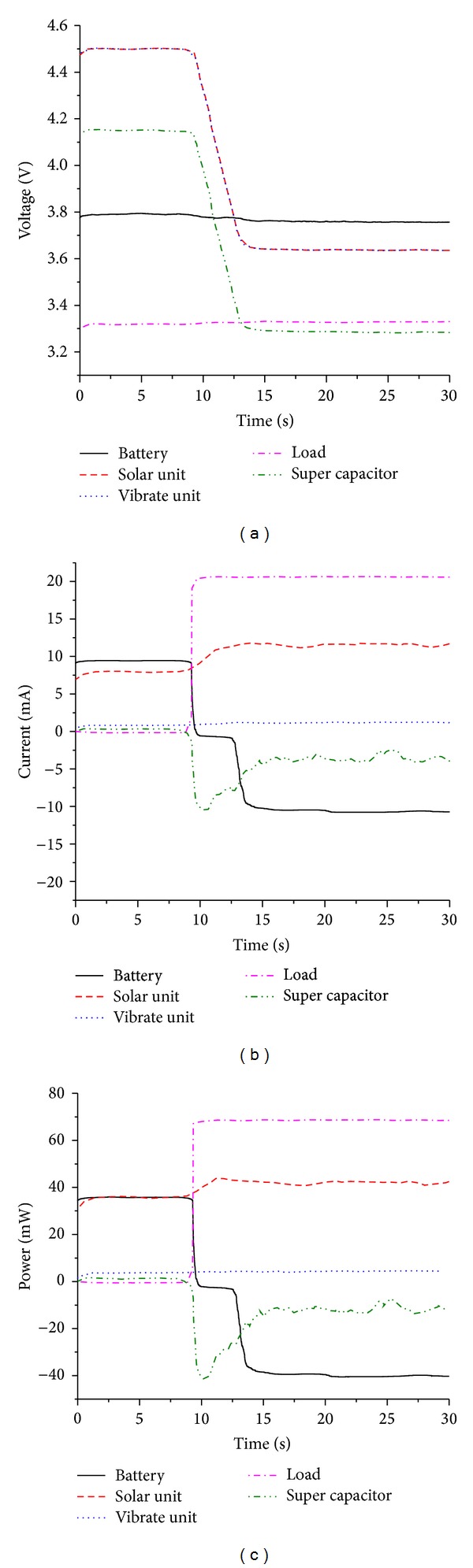
Experiment results with 470 W/m^2^ light power intensity and 1 g amplitude vibration. Negative value in this Figure denotes the discharge current or discharge power of the lithium polymer battery and super capacitor. (a) Voltage curve; (b) current curve; and (c) power curve.

**Figure 12:**
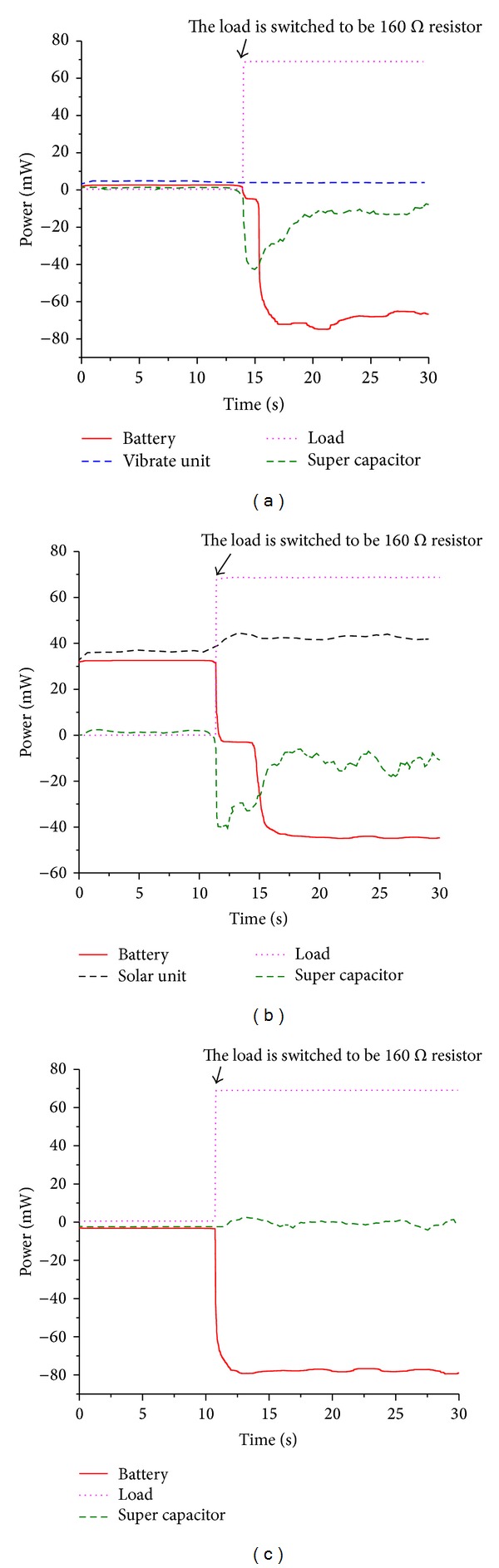
Power curves of the experiment with different ambient energy conditions. Negative value in this figure denotes the discharge power of the lithium polymer battery and super capacitor. (a) Only 1 g amplitude vibration. (b) Only 470 W/m^2^ light power intensity. (c) Without light or vibration.

Figure 10 shows the experimental configuration to evaluate the power management module in different ambient energy conditions. Two resistors (160 Ω and 180 kΩ) are used in this experiment. As the output voltage of the multisource energy harvesting system is 3.3 V, the power of the resistors can be calculated as 68 mW and 60 *μ*W. These values represent the transmission power and sleep power of a typical wireless sensor node [23]. Before the test, the distance between the halogen lamp and the solar panel was adjusted to make sure the light intensity of the solar cell surface is 470 W/m^2^ which represent the average light intensity level in Beijing city in spring and autumn seasons [25]. A 7.8 g mass was placed on the tip of Volture V25W to increase the output power. The shaker was made to vibrate in its resonant frequency (44 Hz) at 1 g amplitude. We define 4 ambient energy conditions for this experiment, which can be listed as follows: (a) 470 W/m^2^ light power intensity and 1 g amplitude vibration, (b) only 470 W/m^2^ light power intensity, (c) only 1 g amplitude vibration, and (d) without light or vibration. For each test, firstly, the 180 kΩ resistor is used to generate low power (the sleep power of WSNs); after several seconds, the load is switched to be 160 Ω resistor to increase the load power (the transmission power of WSNs). Figures 11 and 12 show the results of the experiment.

As shown in Figure 11, when the solar and vibration energy sources are sufficient in the ambient environment, the two energy harvesters work together. At the beginning of the experiment, the output voltage of two energy harvesters is 4.5 V, which is 0.35 V higher than the super capacitor; meanwhile, the voltage of lithium polymer battery is 0.35 V lower than super capacitor. So the Schottky diodes D1, D2, and D3 conduct, and the pMOSFET turns off. The solar energy harvester and vibration energy harvester charge the super capacitor (with 1.5 mW power) and lithium polymer battery (with 35 mW power) and provide the resistor with 60 *μ*W output power.

After about 9 seconds, the load is switched to be 160 Ω resistor. The load power climbs to be 68 mW. Both of the two energy harvesters could not provide such a large power, so the super capacitor begins to discharge immediately. As a result, the voltage of super capacitor decreases to be 3.25 V. Limited by the voltage of super capacitor, the output voltage of solar energy harvester and vibration energy harvester both decrease to be 3.6 V. The voltage difference *V*_gs_ in pMOSFET becomes lower than *V*_th_, so the pMOSFET turns on and lithium polymer battery begins to discharge. From Figure 11(c), we can find that after the load power switching to be 68 mW, the output power of the solar energy harvester increases to about 10 mW. The reason is that lower output voltage of LTC3401 (see Figure 2) results in a smaller voltage difference across it, which increases the convert efficiency of LTC3401 and outputs a larger power.

Figure 12 shows the experiment results when the solar energy harvester and the vibration energy harvester work independently, showing that the power management module successfully realizes its function in controlling the charge and discharge process of the lithium polymer battery according to the load power and ambient energy sources automatically. As shown in Figure 12(a), without the power generating the vibration energy harvester, the charge power of lithium polymer battery becomes lower than the charge power when the two energy harvesters work together (see Figure 11(c)). This confirms that the vibration energy harvester contributes to output power of the system.  Figure 12(b) shows that the vibration energy harvester can power the load and charge the lithium polymer battery independently when the load power is at a low level. If the load power is larger than the power generated by the vibration energy harvester, the lithium polymer battery will discharge to power the load.  Figure 12(c) shows power curves when the solar energy source and vibration energy source are all removed; the lithium polymer battery powers the load independently.

**Figure 13:**
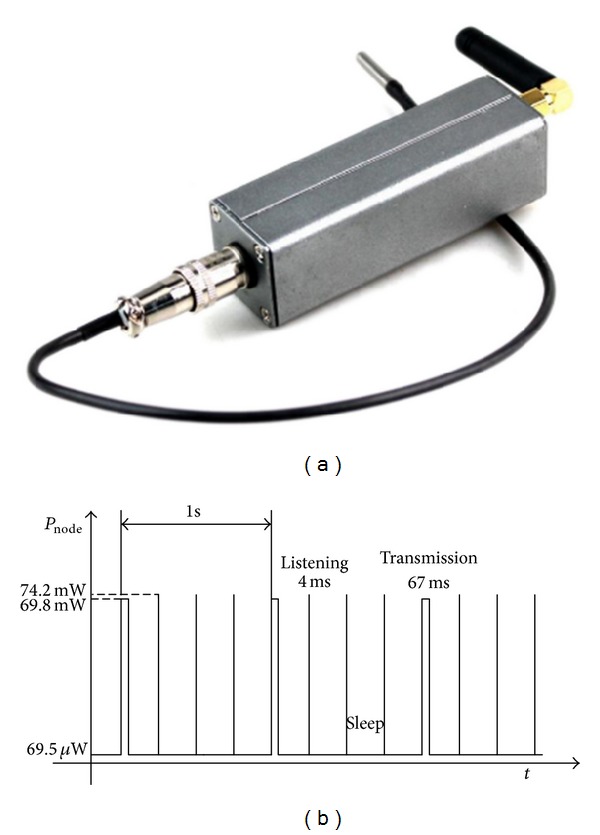
Photo and power consumption of WTHS-G0. (a) Photo of the wireless sensor node WTHS-G0. (c) Power consumption of WTHS-G0 in each state.

From Figure 12, we can find that when the load climbs to a large power abruptly, the super capacitor is prior to lithium polymer battery to discharge. In order to research the role of super capacitor in the multisource energy harvesting system, we did another experiment. For the test, a commercial wireless temperature sensor node WTHS-G0 is used as the load to generate a short periodic pulse power. We make a comparison of the charge power of lithium polymer battery whether the super capacitor exists or not.  Figure 13 shows the photo and power consumption of WTHS-G0. We program it with the monitoring software (see Figure 5) to keep sending data every 1 second.

**Figure 14:**
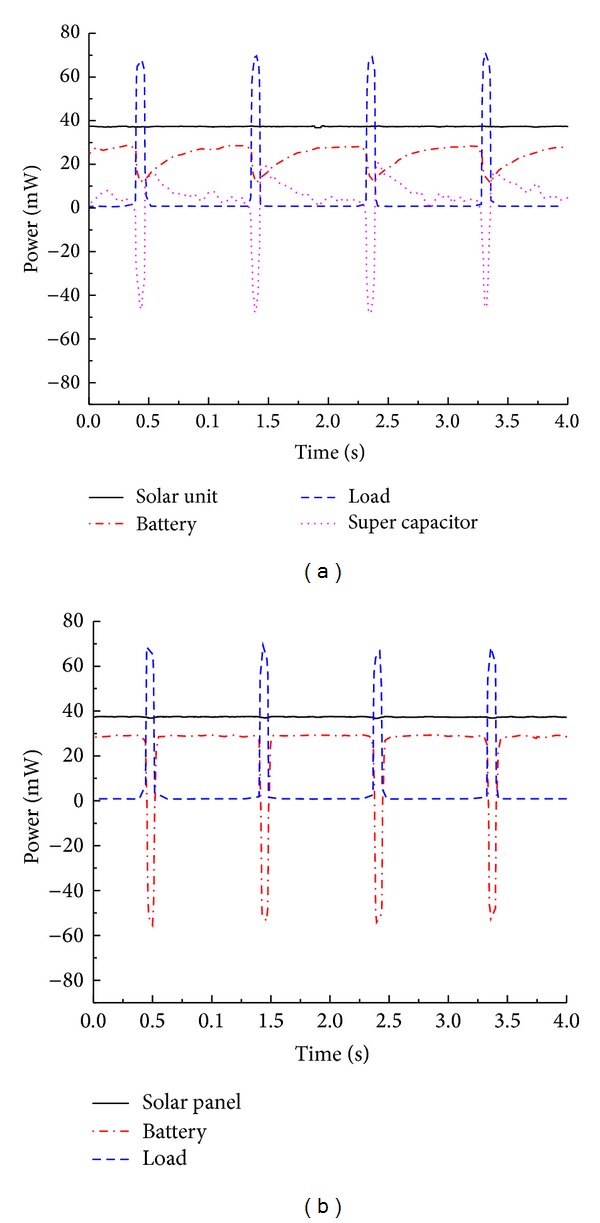
Comparative analysis of the lithium polymer battery charge power. Negative value in this figure denotes the discharge power of the lithium polymer battery and super capacitor. (a) With a 25 mF super capacitor. (b) Without super capacitor.

Figure 14 shows the experiment results. As shown in Figure 14(a), when the pulse power of WTHS-G0 arises, super capacitor is prior to the lithium polymer battery to discharge. Although fluctuation is observed in the charge power of battery, it does not discharge at all.  Figure 14(b) shows the result when we removed the super capacitor. The power of lithium polymer battery fluctuates violently, and it discharges frequently every 1 s. As is known, frequent charge or discharge will shorten the life span of a lithium polymer battery, so the super capacitor can prolong the service life of lithium polymer battery and reduce the current fluctuations.

### 3.5. Prototypes

**Figure 15:**
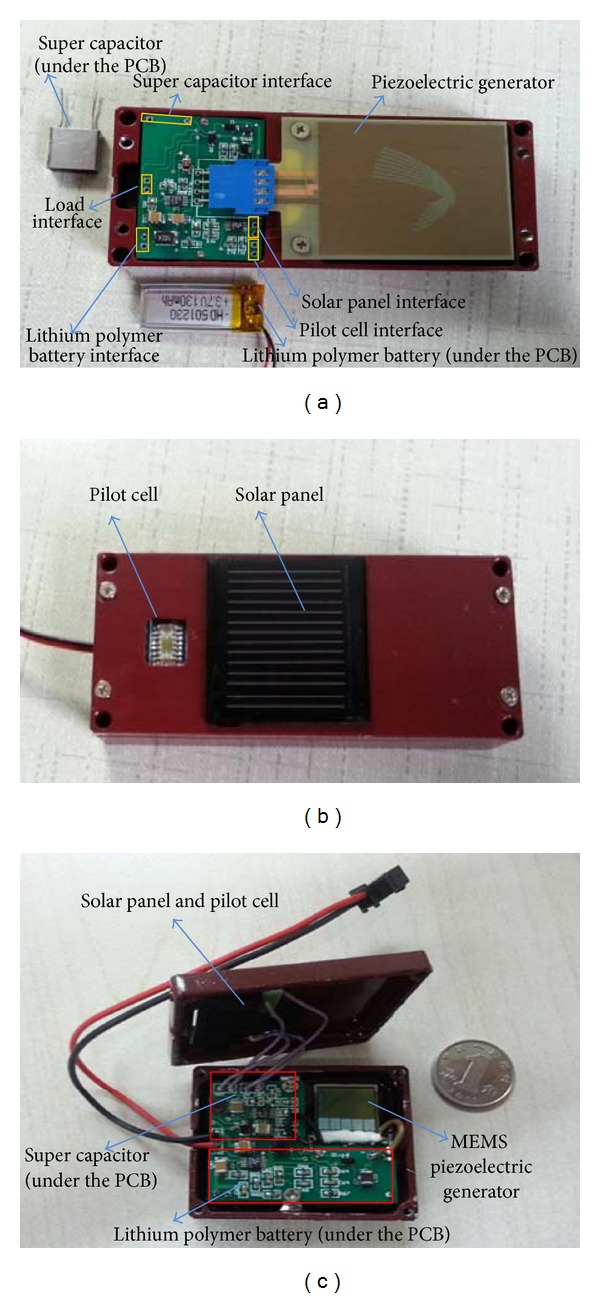
Photos of the multisource energy harvesting system prototypes. (a) Inside photo. (b) Outside photo. (c) Prototype with a MEMS piezoelectric generator.

Based on this research, two prototypes are developed. As shown in Figures 15(a) and 15(b), a small size solar panel and a piezoelectric generator Volture V25W are used as the energy conversion devices in this system. A super capacitor (25 mF) and a lithium polymer battery (3.7 V, 130 mAh) are placed under the circuit board. The overall volume of this prototype is no more than 83 cm^3^. In order to make it easier to integrate the energy harvesting system with wireless sensor node, we replace the Volture V25W with a MEMS piezoelectric generator, the overall volume reduce to 29 cm^3^. The prototypes can provide the WSNs with a 3.3 V constant voltage.

## 4. Environmental Test 

**Figure 16:**
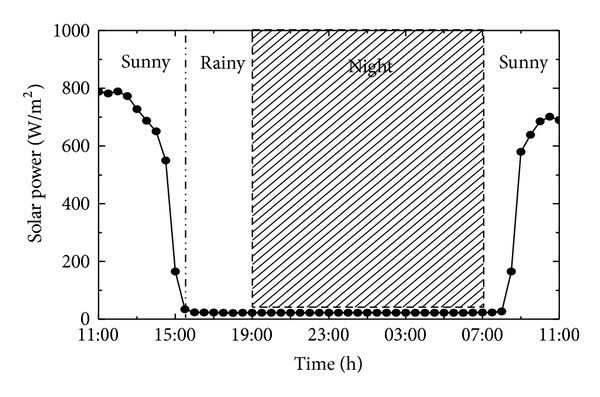
Weather and solar power intensity during the experiment.

In this section, we carry out a 24-hour practical condition test to analyze the output power of the proposed multisource energy harvesting system. The experiment was conducted on an outside balcony in Tsinghua University from 11:00 a.m. (May 1, 2013) to 11:00 a.m. the next day (May 2, 2013). WTHS-G0 node was used for this experiment and was programed to keep sending data every 2 seconds. In this experiment, we did not use a vibration environment, the reason is that the practical vibration in natural environment is a complicated process and has no common criteria to simulate; furthermore, the power generated by the vibration energy harvester is rather smaller than the solar energy harvester when the two energy harvesters work together. It was just used as a supplemental energy source. The weather and solar power intensity in this period are depicted in Figure 16 (measured by OPHIR FL250A-LP1-SH-V1).

**Figure 17:**
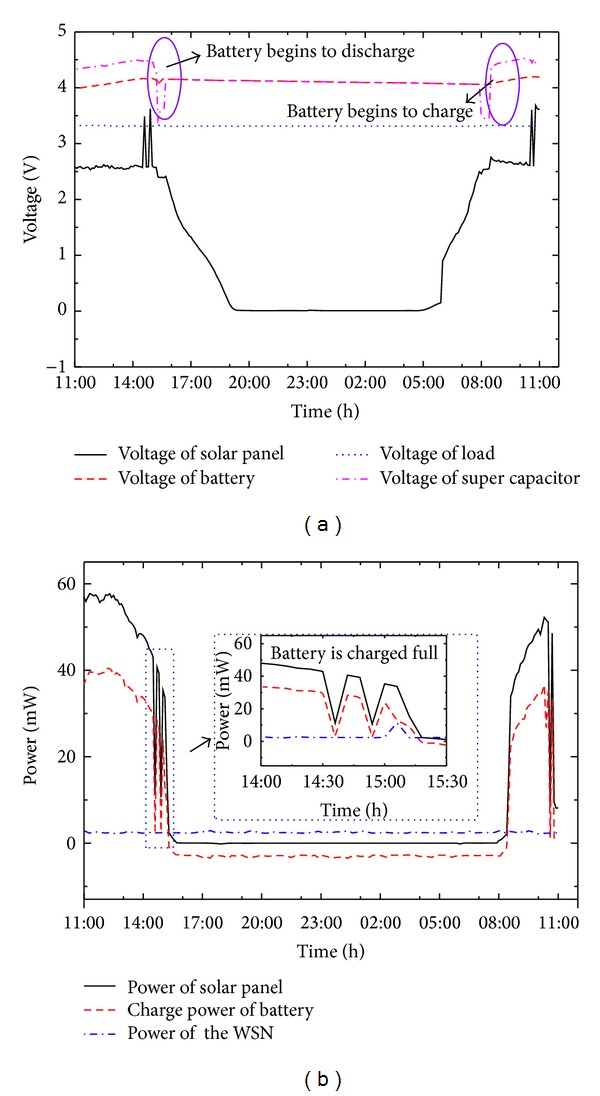
Curves ofvoltage and power in the 24-hour experiment. (a) Voltage curve. (b) Power curve.

Before the test, the lithium polymer battery (130 mAh) was discharged to be drained with an electrochemical workstation (CHI660B) in the mode of constant current discharge at first. In this research, when the battery could not discharge with 1 mA constant current, we define that the state of the battery is empty. Then we charge the battery with 130 mA constant current for 30 min, and define the battery is in initial state. Then, we used the lithium polymer battery to discharge with 130 mA constant current and got a discharge curve of initial state (see Figure 19) and start the experiment. For this experiment, we used MAX4071 to measure the currents and AMUSB-9110 to collect experiment data. We recorded the voltages and currents of the solar panel, lithium polymer battery, super capacitor, and WTHS-G0.  Figure 17 shows the experimental data collected over 24 hours.

During the period from 11:00 to 14:00, the weather was sunny and the lithium polymer battery was in the state of charging. The power curve of solar panel and the curve of lithium polymer battery charge power were varying with the solar power intensity. In the period from 14:00 to 15:30, the charge power of battery was decreasing and fluctuating. The reason is that the battery is charged full and the voltage of the battery climbed to the peak value of 4.17 V. The protecting circuit inside the lithium polymer battery cut down the charge circuit. So the charging power of battery decreased. After an internal charge-balancing process in the lithium polymer battery, the voltage decreases below 4.17 V and the battery started to charge again, so the curve featured jagged. At 15:30, the weather got to be rainy and light disappeared. At this moment, the super capacitor discharged to power the node at once. After 10 min, super capacitor could not power the node anymore, and the lithium polymer battery began to discharge. During the period from 19:00 to 7:00, the WTHS-G0 node was powered by the lithium polymer battery. At 8:00, the sun came out and the battery began to charge again.

**Figure 18:**
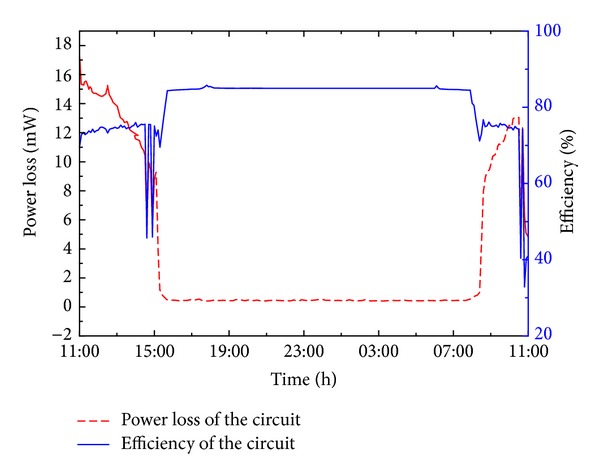
Power losses in the energy harvesting system and the overall efficiency in the 24-hour experiment.

Figure 18 shows the power loss in the energy harvesting system and the overall efficiency. In this figure, the power loss is defined in (5), where *P*_loss_ denotes the power loss in the energy harvesting system. *P*_load_, *P*_pv_, and *P*_batt_, respectively, denote the power of WTHS-G0 node, power of solar cell, and power of lithium-polymer battery. Consider

(5) $$\begin{array}{c} P_{\text{loss}}=P_{\text{pv}}-\left(P_{\text{node}}+P_{\text{batt}}\right). \end{array}$$

To calculate the efficiency of the circuit, we should consider the state of the lithium-polymer battery. When the battery is in the state of charge (*P*_batt_ is positive), the efficiency of the circuit can be expressed in (6). If the battery is in the state of discharge (*P*_batt_ is negative), the efficiency should be expressed in (7). Consider

(6) $$\begin{array}{c} \eta_{\text{circuit}}=\frac{\left(P_{\text{node}}+P_{\text{batt}}\right)}{P_{\text{pv}}}, \end{array}$$

(7) $$\begin{array}{c} \eta_{\text{circuit}}=\frac{P_{\text{node}}}{\left(P_{\text{pv}}-P_{\text{batt}}\right)}. \end{array}$$

As shown in Figure 18, during the period of light radiation time, the power losses in the system are at the range from 10 mW to 15 mW, which is caused by the LTC3401 converter in the solar energy harvester and LTC 3532 converter in power management circuit. When the light disappears in the night, the power losses bellow 0.4 mW and are only caused by the power management circuit. The overall efficiency of the energy harvesting circuit is at the range from 75% to 85%. In the periods from 14:00 to 15:30 and 9:00 to 11:00 the next day, the efficiency is violently fluctuating; the reason is that the fully charged state of lithium-polymer battery resulted in the solar energy harvester not working in the MPPT mode, so the efficiency decreased at that moment. After the lithium-polymer battery got into the state of charge, the solar energy harvester started to work in the MPPT mode again, and the efficiency of the circuit increased.

**Figure 19:**
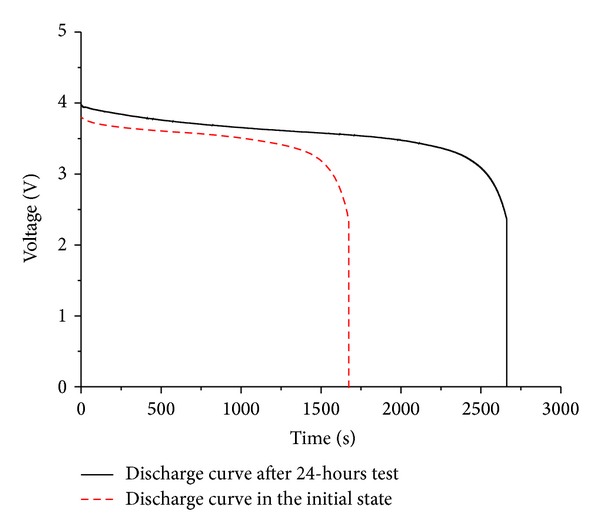
Discharge curve of lithium polymer battery with 130 mA constant current.

After the test, we used CHI660B electrochemical workstation to discharge the battery with 130 mA to get another discharge curve and make a comparison between the two curves. As shown in Figure 19, the initial energy stored in the battery can be calculated as 755.82 J. After a 24-hour test, the energy increases to be 1231.38 J, showing that the power generated by the energy harvesting system is much more than the energy consumed by the node, and 476 J energy was storied in the lithium polymer battery.

## 5. Conclusion

In this paper, we have presented an effective multisource energy harvesting system. It harvests energy from multiple ambient power sources (solar and vibration energy) autonomously and simultaneously while performing MPPT on each power source. Its fully analog design based on ultra-low-power components makes it a very efficient and cost-effective solution to enable the autonomous operation of WSNs. The power management circuit permits to harvest ambient energy from an expandable number of different sources and realizes power switch smartly and quickly. The design of energy reservoirs using lithium polymer battery ensures a continuous power supply to the load system even during long periods of ambient energy shortage. The connected super-capacitor provides WSNs with a large instantaneous output power and protects the battery from charging and discharging frequently. Based on these works, prototypes have developed and we integrate an MEMS piezoelectric generator for vibration energy harvesting in the multisource energy harvesting system for the first time in literature. With the MEMS piezoelectric generator, the overall volume can be reduced to 29 cm^3^, which makes it possible to be integrated with WSN. Experimental results through the 24-hour environmental test show that the proposed multisource energy harvesting system is effective and reliable and can be used as a continuous power supply for low power WSNs.
